# Heat Shock Protein 90α Is a Potential Serological Biomarker of Acute Rejection after Renal Transplantation

**DOI:** 10.1371/journal.pone.0162942

**Published:** 2016-09-15

**Authors:** Takeshi Maehana, Toshiaki Tanaka, Hiroshi Kitamura, Nobuyuki Fukuzawa, Hideki Ishida, Hiroshi Harada, Kazunari Tanabe, Naoya Masumori

**Affiliations:** 1 Department of Urology, Sapporo Medical University School of Medicine, Sapporo, Japan; 2 Department of Kidney Transplant Surgery, Sapporo City General Hospital, Sapporo, Japan; 3 Department of Urology, Tokyo Women’s Medical University, Tokyo, Japan; University of Toledo, UNITED STATES

## Abstract

**Background:**

Heat shock protein 90 (HSP90), a molecular chaperone associated with the activation of client proteins, was recently reported to play an important role in immunologic reactions. To date, the role of HSP90 in solid organ transplantations has remained unknown. The aim of this study was to evaluate the relationship between serum HSP90α levels and acute allograft rejection after organ and tissue transplantation using serum samples from kidney allograft recipients, an in vitro antibody-mediated rejection model, and a murine skin transplantation.

**Results:**

Serum HSP90α levels were significantly higher in kidney recipients at the time of acute rejection (AR) than in those with no evidence of rejection. In most cases with AR, serum HSP90 decreased to baseline after the treatment. On the other hand, serum HSP90α was not elevated as much in patients with chronic rejection, calcineurin inhibitor nephrotoxicity, or BK virus nephropathy as in AR patients. In vitro study showed that HSP90α concentration in the supernatant was significantly higher in the supernatant of human aortic endothelial cells cocultured with specific anti-HLA IgG under complement attack than in that of cells cocultured with nonspecific IgG. In mice receiving skin transplantation, serum HSP90α was elevated when the first graft was rejected and the level further increased during more severe rejection of the second graft.

**Conclusions:**

The results suggest that HSP90α is released into the serum by cell damage due to AR in organ and tissue transplantation, and it is potentially a new biomarker to help detect AR in kidney recipients.

## Introduction

The development of immunosuppressive treatments has decreased the incidence of acute rejection (AR) and improved the outcome in solid organ transplantation. However, careful surveillance for detection of AR is still mandatory in most allograft recipients. In kidney transplantation, serum creatinine is currently used as the sole serological marker of acute rejection, but it is also elevated due to other conditions such as infectious disease and drug nephrotoxicity. Thus, histological diagnosis by graft biopsy is essential to start anti-acute rejection treatment. However, graft biopsy is invasive and associated with hemorrhagic complications. On the other hand, kidney transplant recipients often have a concomitant cardiovascular disease or cerebrovascular disease needing anticoagulation or antiplatelet therapy. Because cessation of such therapy is necessary from several days before biopsy, the diagnosis of rejection can be delayed. Moreover, cessation of anticoagulation or antiplatelet therapy may involve a high risk of recurrence or worsening of the cardiovascular or cerebrovascular disease. In addition, the cooperation of the patient is mandatory for graft biopsy. Therefore, mental disorders such as delirium, and an uncooperative attitude may make it difficult to carry out graft biopsy. Furthermore, severe AR such as antibody-mediated rejection is time-sensitive and requires quick initiation of the treatment to restore the graft function. Therefore, a highly specific serological marker for AR would be quite helpful in the clinical setting of kidney transplantation.

Heat shock protein 90 (HSP90) is a molecular chaperone of 90KDa and a constitutively expressed cellular protein that compromises 1%–2% of the total protein load [1]. Recently, there have been reports on its importance in immunologic reactions. HSP90 has been shown to play important roles in antigen presentation, activation of lymphocytes and macrophages, maturation of dendritic cells, and in the enhanceosome-mediated induction of inflammation [2]. Moreover, serum HSP90 is associated with activity in some autoimmune diseases. Free HSP90 is released into the sera of patients with active systemic lupus erythematosus (SLE) [3], whereas it is reduced in those with bullous pemphigoid [4]. Although, to date, there is no report showing its role in allograft rejection, we hypothesized that HSP90 was potentially involved in the alloresponse after solid organ transplantation and that the serum level of HSP90 would potentially be influenced by allograft rejection. The aim of this study was to evaluate the relationship between serum HSP90α levels and acute allograft rejection after solid organ transplantation using serum samples from kidney allograft recipients, an in vitro antibody-mediated rejection model, and a murine skin transplantation model.

## Materials and Methods

### Patients

We obtained 96 serum samples from 70 patients who underwent kidney transplantation at the Sapporo Medical University Hospital, Sapporo City General Hospital, and Tokyo Women’s Medical University Hospital. This study was reviewed and approved by the Ethical Committee of Sapporo Medical University School of Medicine, Sapporo City General Hospital and Tokyo Women’s Medical University (Representative Institutional Review Board No. 262–102) and was conducted in accordance with the Declaration of Helsinki. All the patients were invited voluntarily after a clear explanation about the study objectives. All participants or next of kin provided written informed consent that was freely given. None of the transplant patients were from a vulnerable population. All the patient’s records were anonymized by giving a number to each sample before the analysis. We published the commencement of this study on our website (http://web.sapmed.ac.jp/uro/) and presented that the patients who participated in this study can refuse later.

All patients received immunosuppressive treatment consisting of induction therapy using basiliximab, a calcineurin inhibitor (CNI, cyclosporine (CsA) or tacrolimus (TAC)), mycophenolate mofetil (MMF) and a steroid and following maintenance therapy using the CNI (CsA or TAC), MMF and a steroid. Sensitized patients underwent desensitization treatment including plasmapheresis, intravenous immunoglobulin and anti-CD20 mAb administration at the time of transplantation. Patients who exhibited rejection from 2009 to 2014 were chosen deliberately. Cases with no evidence of rejection were chosen at random. Thus, this study does not include all transplanted cases in this period. All patients underwent protocol biopsy at 3–6 months after transplantation and annually thereafter or indication biopsy when rejection was suspected. Serum samples were collected when protocol and indication graft biopsies were conducted. These samples were classified according to histology that was proven by biopsy at that time. For patients with acute rejection proven by biopsy, serum samples were taken after completion of anti-rejection treatment and graft biopsy was done to confirm resolution of the rejection. No patients had complications in the circulatory or respiratory system. At the diagnosis of rejection, they had no severe clinical condition such as dyspnea, high fever, sepsis or shock. The clinicopathological characteristics of the patients in each group except for those with no evidence of rejection and other disease impairing renal function are shown in the Table 1. The type of rejection was classified according to the 2009 Banff criteria [5]. Thirsty-seven patients had no evidence of rejection and other disease. Acute T-cell-mediated rejection (ATMR), acute antibody-mediated rejection (AABMR) and chronic antibody-mediated rejection (CABMR) were diagnosed histopathologically in 8 patients each. Calcineurin inhibitor nephrotoxicity (CIN) and BK virus nephropathy (BKVN) were diagnosed in 5 and 4 patients, respectively. The diagnosis of CIN was based on histopathologic examination of renal biopsy specimens and detection of a high concentration of a calcineurin inhibitor in the serum. Histopathological findings suggestive of renal toxicity of CNI include isometrical tubular epithelial vacuolization in renal tubular epithelial cells mainly in the acute phase and microcalcification or vascular changes in the chronic phase [5]. In this study, all CIN was induced by TAC. The dosage was controlled according to the trough level in the blood. In principle, CIN was diagnosed when the biopsy confirmed the histopathological findings suggestive of CIN as well as a trough level of blood TAC > 8 ng/ml in the first 1 year and > 5 ng/ml thereafter, which were considered compatible with CIN. In this study, 5 cases met these criteria for CIN. The diagnosis of BKVN was based on histopathologic examination of renal biopsy specimens and detection of BKV DNA in the plasma and urine by polymerase chain reaction assay. When decoy cells were detected in urine cytology for outpatients, we checked for BKV DNA in the plasma and urine. Values of more than 10^7^ BKV copies per milliliter obtained in serum or urine samples repeatedly were considered indicative of BKV infection. Immunohistochemical staining of biopsy specimens for simian virus 40 large T antigen was done for the definitive diagnosis of BKVN [6]. Moreover, all serum samples were obtained one month or more after the transplantation. The sera were divided into aliquots after centrifugation and stored at -80°C. After the event of rejection, a drop in the serum creatinine level and improvement of histological findings were considered to indicate recovery from AR. Serum levels of HSP90α were measured by enzyme-linked immunosorbent assay (ELISA) according to the manufacturer’s instructions (ADI-EKS-895, Enzo Life Sciences, USA).

**Table 1:** Clinicopathological characteristics of the patients.

Pathological finding	No	Sex	Age at diagnosis (years)	Time after KTx (months)	Type of biopsy	Cre (ml/dl)	DSA	Banff type	sHSP90α at rejection (ng/ml)	Treatment	sHSP90α after resolution of AR (ng/ml)
ATMR	1	male	47	21	Indication	1.31	negative	IA	13.84	steroid pulse	7.49
	2	male	65	6	Protocol	1.21	negative	IB	37.04	steroid pulse, deoxyspergualin	8.41
	3	male	36	3	Protocol	1.78	negative	IB	10.41	steroid pulse, deoxyspergualin	19.39
	4	male	35	4	Indication	2.52	negative	IB	36.71	steroid pulse, deoxyspergualin	19.30
	5	male	46	3	Protocol	1.80	negative	IB	16.70	steroid pulse	11.93
	6	male	63	24	Protocol	1.50	negative	IB	9.92	steroid pulse	7.65
	7	male	58	3	Protocol	1.17	negative	IIA	44.81	steroid pulse, deoxyspergualin	19.84
	8	male	40	14	Indication	1.65	negative	IIB	53.69	steroid pulse, deoxyspergualin	11.29
AABMR	1	male	30	4	Indication	2.43	positive	I	40.17	steroid pulse, deoxyspergualin, muromonab-CD3	N.A.
	2	male	56	7	Indication	1.98	positive	I	60.17	steroid pulse, deoxyspergualin, muromonab-CD3	4.40
	3	male	29	20	Indication	2.39	positive	I	16.20	steroid pulse, deoxyspergualin, muromonab-CD3	23.60
	4	female	47	2	Indication	1.00	positive	I	17.41	steroid pulse, deoxyspergualin	4.78
	5	female	53	7	Indication	1.98	positive	I	14.79	steroid pulse, deoxyspergualin, immunoglobulin	2.17
	6	male	38	2	Indication	2.53	positive	I	15.07	steroid pulse, rituximab	N.A.
	7	male	65	5	Indication	1.61	positive	II	58.98	steroid pulse, deoxyspergualin, plasma exchange	3.85
	8	male	48	12	Indication	2.80	positive	II	68.60	steroid pulse, deoxyspergualin, plasma exchange, rituximab	7.92
CABMR	1	male	45	30	Indication	1.74	positive	-	6.81	Increase MMF dosage	-
	2	male	28	108	Indication	1.38	positive	-	2.55	Increase MMF dosage	-
	3	female	50	26	Indication	0.95	positive	-	0.31	Increase MMF dosage	-
	4	male	59	72	Protocol	1.41	positive	-	12.89	Increase MMF dosage	-
	5	male	35	146	Indication	2.29	positive	-	2.04	Increase MMF dosage, deoxyspergualin, immunoglobulin	-
	6	female	48	24	Protocol	1.16	positive	-	12.54	Increase MMF dosage	-
	7	male	36	18	Indication	1.33	positive	-	1.53	deoxyspergualin	-
	8	female	56	27	Indication	0.75	positive	-	4.05	deoxyspergualin	-
CIN	1	male	66	24	Protocol	0.89	negative	-	4.51	Reduce TAC dose, Add EVR	-
	2	male	39	122	Protocol	2.00	negative	-	6.96	Switching from TAC to TACER, Reduce TAC dose	-
	3	male	67	60	Protocol	1.11	negative	-	7.54	Reduce TACER dose	-
	4	male	66	45	Protocol	1.44	negative	-	4.59	Reduce TACER dose	-
	5	male	26	8	Indication	1.69	negative	-	9.73	Reduce TACER dose	-
BKVN	1	male	63	11	Indication	1.86	negative	-	17.23	Reduce TAC and MMF doses, cidofovir	-
	2	male	48	3	Indication	3.08	negative	-	6.43	Reduce TAC and MMF doses, cidofovir	-
	3	male	51	18	Indication	2.00	negative	-	12.73	Reduce TAC and MMF doses, cidofovir	-
	4	male	48	63	Indication	1.20	negative	-	7.39	Reduce TAC and MP doses	-

ATMR, acute T cell-mediated rejection; AABMR, acute antibody-mediated rejection; CABMR, chronic antibody-mediated rejection; CIN, calcineurin inhibitor nephrotoxicity; BKNV, BK virus nephropathy; KTx, kidney transplantation; Cre, creatinine; DSA, donor-specific antibody; sHSP90α, serum heat shock protein 90α; MMF, mycophenolate mofetil; TAC, tacrolimus; TACER, tacrolimus extended-release; MP, methylprednisolone; N.A., not applicable.

### In vitro antibody-mediated rejection (ABMR) model

In this study, we created an in vitro human alloantibody-mediated rejection model. Human aortic endothelial cells (HAEC) were purchased from the American Type Culture Collection [Primary Aortic Endothelial Cells, PCS-100-011, Lot #58570433, American Type Culture Collection (ATCC), USA]. The cells were cultured in Vascular Cell Basal Medium (ATCC, USA) using an Endothelial Cell Growth Kit-VEGF (ATCC, USA). The HLA genotype of HAEC was determined using a polymerase chain reaction sequence-based typing method. A highly sensitized serum sample was obtained from a patient who had HAEC-specific anti-HLA antibodies. These antibodies were confirmed with Luminex assays (Luminex, USA). An unsensitized serum sample was then obtained from a healthy volunteer with type AB blood. IgG was purified from the serum samples using a Melon Gel Spin Purification Kit (Thermo Scientific, USA).

For the immunofluorescence analysis, cells were cultured with IgG that had been purified from sensitized serum on BD Biocoat Fibronectin 4-well CultureSlides (BD Biosciences, USA). As a control, cells were incubated with unsensitized IgG. These incubations were conducted for 12 h in growth-factor-free-medium. IgG was identified using TRITC-conjugated donkey anti-human IgG with 4, 6-diamidino-2-phenylindole (DAPI) for nuclear staining. After the staining, the slides were washed and fluorescent immunohistochemical analysis was performed with a Keyence Biorevo BZ-9000 microscope (Keyence, USA). For flow cytometry, HAECs were cultured with specific or unspecific IgG for 12 h in growth-factor-free medium. After incubation, the cells were fixed and permeabilized using a FlowSelect cell signaling kit (Millipore, Germany), and stained with FITC-labeled geldanamycin (StressMarq Biosciences, Canada). Flow cytometric analysis of the stained cells was performed using an Attune Acoustic Focusing Cytometer (Applied Biosystems, Thermo Fisher Scientific, USA).

We also assessed the complement-mediated cytotoxicity (CMC) in this model. The cells were preincubated in 24-well culture plates (BD Biosciences, USA) for 24 h in growth-factor-free medium. After the preincubation, both sensitized and unsensitized IgG were added to each well, followed by incubation for an additional 24 h. Finally, complement (standard rabbit complement, Cedarlane, Canada) was added to each well and it was incubated for 30 more minutes. The HSP90α concentrations of the supernatants were measured in each well using ELISA (ADI-EKS-895, Enzo Life Sciences, USA).

### Skin transplantation of animal model

Mice were cared for in accordance with the Principles of Laboratory Animal Care (NIH publication 86–23, revised 1985). This project was submitted to and approved by the Institutional Animal Care and Use Committee of Sapporo Medical University (protocol No. 14–035). Female C57BL/6 (H-2b) and BALB/c (H-2d) mice (6–8 weeks old, weighing 18–22 g) were obtained from Hokudo Co., Ltd., (Sapporo, Japan). We used 66 BALB/c and 40 C57BL/6J mice. Animals were held under standard conditions with unlimited access to water and standard laboratory food and were housed for at least 1 weeks under special pathogen free conditions prior to transplantation. All surgery was performed under isoflurane anesthesia, and all efforts were made to minimize suffering. We monitored the health of the animals every day after transplantation and there were no unexpected deaths in this model.

Fully-mismatched skin transplantation was performed using the C57BL/6 mice as donors and the BALB/c mice as recipients. A square full thickness back skin graft (1.5 × 1.5 cm) was placed on a graft bed that had been prepared on the back of each recipient mouse [7]. The graft was covered with protective bandages for 6 days. The transplanted graft was visually checked daily after removing the bandages and was considered fully rejected when 90% of it was necrotic. Syngeneic transplantation (H-2d to H-2d) was used as a control. In the AABMR model, transplantations were performed again 14 days after the first transplantations. The protective bandages were removed at 4 days and the skin grafts were visually checked daily. Serum samples were collected on days 0, 7, at the time of rejection and on day 14 after the first transplantation and on days 0, 4 and at the time of rejection after the second transplantation. In the syngeneic group, serum samples were collected on days 0, 7, 14, and 21. Mice were humanely euthanized by cervical dislocation or via overdose with an intraperitoneal injection of sodium pentobarbitone anesthetic, according to standardized protocols supplied by the Sapporo Medical University at the time of collecting blood or skin graft. After animals were anesthetized and sacrificed, blood samples were immediately collected through cardiac puncture and centrifuged at 3000 rpm for 15 minutes, and then the serum was removed and frozen at -80°C until they were assayed. Serum HSP90α was analyzed by ELISA according to the manufacturer’s instructions (CSB-E08312M, Cusabio Biotech, USA). Donor-specific antibodies (DSA) were detected by flow cytometry as follows [8]. Aliquots of donor-strain thymocyte suspensions were incubated with serial dilutions of pretransplant recipient sera and at several time points between transplantation and rejection. After 30 min, the cells were washed and stained with FITC-conjugated goat anti-mouse IgG, and an Fcγ fragment-specific mAb (Jackson Immunoresearch, West Grove, PA, USA). The mean channel fluorescence (MCF) of each dilution of each serum sample was determined and the dilution that returned the MCF to the level observed when BALB/c thymocytes were stained with a 1:4 dilution of naive serum was divided by two and reported as the titer. For histological analyses, after animals were anesthetized and sacrificed, skin grafts were removed at day 6 after transplantation in each group and immediately fixed in 10% formaldehyde. Following this, the specimens were embedded in cassettes with paraffin. Then 3μm sections of the formalin-fixed, paraffin embedded tissues were stained with hematoxylin and eosin.

### Statistical analysis

All in vitro experiments were repeated at least three times. The results were given as median and mean ± SD. Comparisons between two groups were performed using the Mann-Whitney U test and the Wilcoxon signed-rank test and those for three groups were performed using the Tukey's multiple comparison test. Correlation analysis was performed using Spearman rank correlation. P values of less than 0.05 were considered statistically significant. All statistical analyses were performed with EZR statistical software (Saitama Medical Center, Jichi Medical University) [9], which is a graphical user interface for R (The R Foundation for Statistical Computing, version 2.13.0). More precisely, it is a modified version of R Commander (version 1.6–3) that includes the statistical functions that are frequently used in biostatistics.

## Results

### Serum HSP90α was elevated at the time of acute rejection after renal transplantation

**Fig 1:**
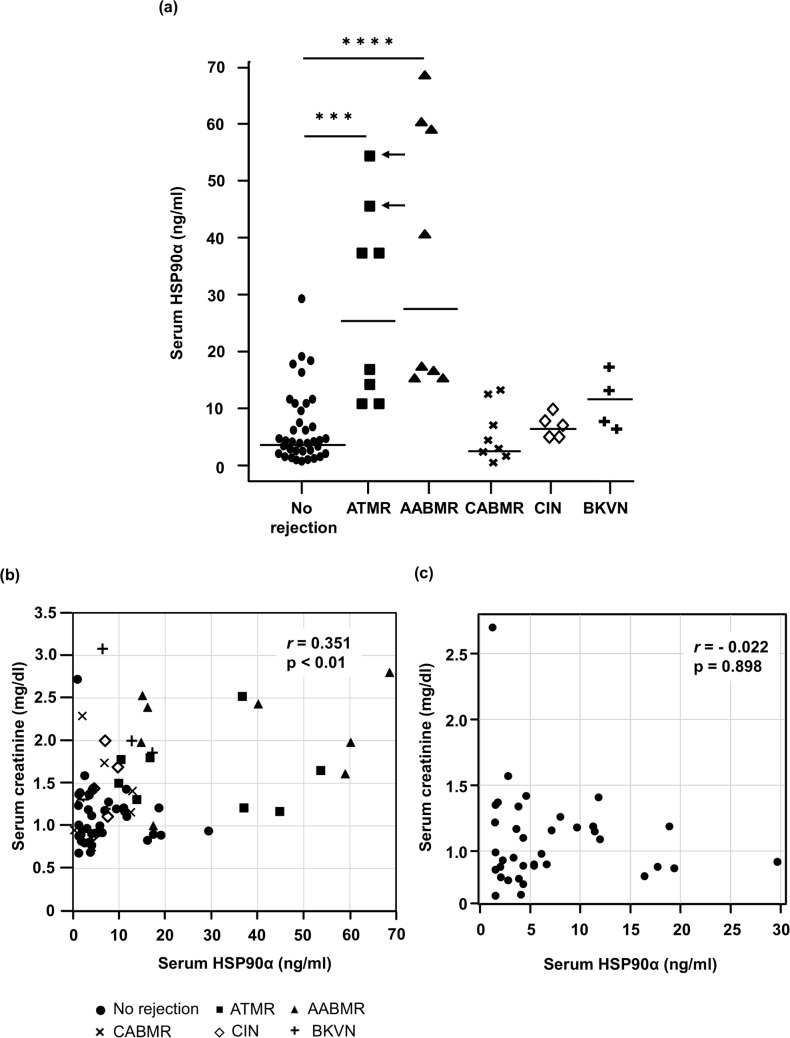
Scatter plots of serum HSP90α and serum creatinine in kidney recipients. Serum HSP90α was measured simultaneously with serum creatinine in kidney recipients with various conditions. Each dot shows a single sample from a single patient according to the graft status, and the bars represent the median. (a) Serum HSP90α levels were significantly higher at the time of acute T cell-mediated rejection (ATMR) and acute antibody-mediated rejection than for those with no evidence of rejection. The arrows indicate samples at the time of Banff Type II ATMR. (b) Correlation diagram of serum HSP90α and serum creatinine in all cases. (c) There was no significant correlation between serum creatinine and HSP90α serum levels in 37 cases with no evidence of rejection. ***p < 0.001, ****p < 0.0001. ATMR, acute T cell-mediated rejection; AABMR, acute antibody-mediated rejection; CABMR, chronic antibody-mediated rejection; CIN, calcineurin inhibitor nephrotoxicity; BKVN, BK virus nephropathy.

**Fig 2:**
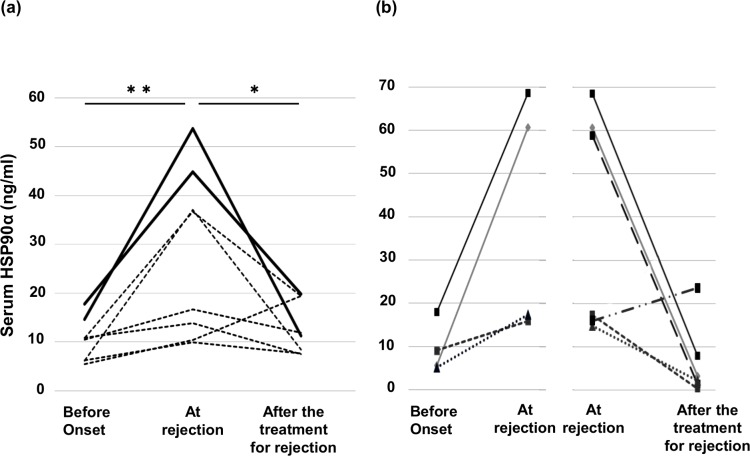
**Kinetics of serum HSP90α before and after acute T-cell-mediated rejection (a) and acute antibody-mediated rejection (b).** In most patients, serum HSP90α levels increased after the onset of acute rejection compared to those before onset and decreased to baseline after treatment. (a) Results of serial measurement of serum HSP90α in eight patients with acute T-cell-mediated rejection. Patients with Banff type II (black line) had a higher level at rejection than those with type I (dotted line). (b) Serum HSP90α was measured in four patients before onset and at rejection and was measured in six patients at rejection and after treatment. Values after treatment in the former four patients and baseline values in the latter six patients were not available. *p < 0.05, **p < 0.01

The serum HSP90α levels in kidney recipients are shown in Fig 1A. The median serum HSP90α levels at the times of ATMR and AABMR were 26.71 ng/mL and 28.79 ng/mL, respectively, which were significantly higher than those at the time with no evidence of rejection (4.05 ng/mL, p < 0.001 and p < 0.0001, respectively). In contrast, the serum HSP90α levels were not elevated in other conditions such as CABMR and CIN (median 3.30 ng/mL and 6.96 ng/mL, respectively). The median HSP90α concentration of BKVN patients was 10.06 ng/mL, which was a 2.5-fold increase compared to the cases with no evidence of rejection. Serum HSP90α levels > 30 ng/ml were found only in serum samples from recipients with vasculitis in the graft such as during AABMR and Banff type II ATMR. The serum creatinine level in each condition and a correlation diagram for HSP90α are shown in Fig 1B. There was a weak, but significant correlation between the serum creatinine and HSP90α levels in the entire series (*r* = 0.351, p < 0.01). However, there was no significant correlation between the serum creatinine and HSP90α levels in the no-rejection group (r = -0.022, p = 0.898, Fig 1C) or the other groups (data not shown). Moreover, using the Wilcoxon signed-rank test, we observed that the serum HSP90α level in ATMR patients significantly decreased and that in AABMR patients nearly significantly decreased to baseline levels after the treatment for rejection (p < 0.05 and p = 0.062, respectively, Fig 2).

### HSP90α was released in the supernatant in an in vitro ABMR model

**Fig 3:**
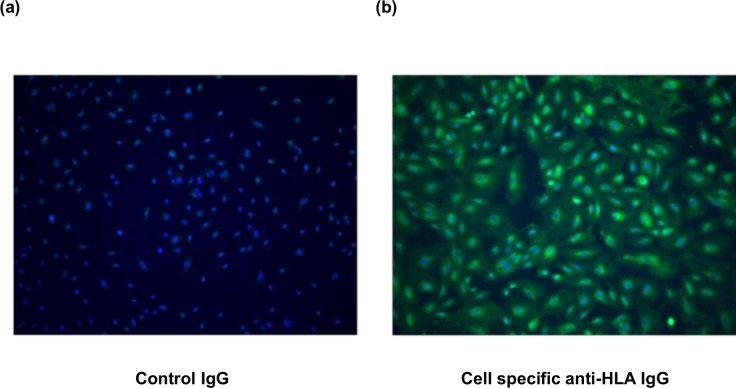
Purified IgG was specifically ligated to human aortic endothelial cells in immunofluorescence analysis. (a) Human aortic endothelial cells (HAEC) were cultured within the control IgG. Nonspecific IgG is not ligated to the HAEC. (b) HAEC were incubated within a specific anti-HLA IgG. Purified IgG is specifically ligated to the HAEC. Pseudo-green: anti-human IgG, pseudo-blue: DAPI.

**Fig 4:**
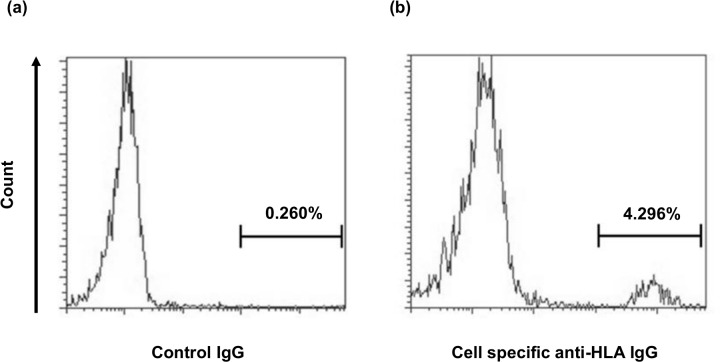
Surface expression of HSP90α was enhanced by binding of the anti-HLA antibody. Human aortic endothelial cells were incubated within the specific anti-HLA IgG or nonspecific IgG. After incubation, the cells were stained with FITC-labeled geldanamycin and analyzed. Flow cytometry shows increased expression of geldanamycin-positive cells with anti-HLA IgG ligation (b, 4.296%) versus control IgG (a, 0.460%). This experiment was independently performed three times and the results obtained were substantially the same.

**Fig 5:**
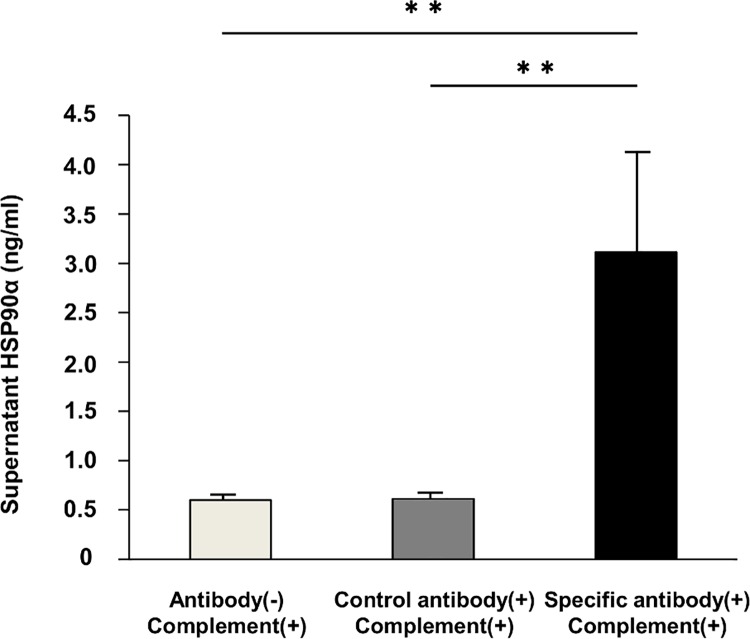
The HSP90α concentration in the supernatant of human aortic endothelial cells under complement attack. Levels of HSP90α in the supernatants of human aortic endothelial cells (HAEC) in three groups were measured by ELISA. Bars show mean ± SD from three independent experiments. Statistically significant differences were observed between the subgroups of HAEC that were cocultured with anti-HLA IgG and cocultured with nonspecific IgG. **p < 0.01.

We created an in vitro ABMR model using HAEC and a HAEC-specific anti-HLA antibody. The profile of the HLA haplotype of HAEC was A*02:01-C*05:01-B*44:02-DRB1*04:01-DQB1*03:01-DPB1*04:01 and we purified IgG from the serum of a patient with an anti-B44 antibody. In immunofluorescence analysis, the purified IgG was specifically ligated to HAEC, whereas the nonspecific IgG was not ligated to HAEC (Fig 3). Moreover, the geldanamycin-positive cells were increased after anti-HLA IgG ligation compared with the controls in flow cytometric analysis (Fig 4). This result indicated that anti-HLA IgG ligation increased the number of HSP90-positive cells because geldanamycin specifically binds to HSP90α [10]. The HSP90α concentration in the supernatant of HAEC that were cocultured with anti-HLA IgG under complement attack was significantly higher than that in the supernatant cocultured with nonspecific IgG (3.11 ± 1.02 ng/mL vs. 0.61 ± 0.02 ng/mL, p < 0.01, Fig 5)

### Serum HSP90α was elevated at the time of rejection in skin transplantation

**Fig 6:**
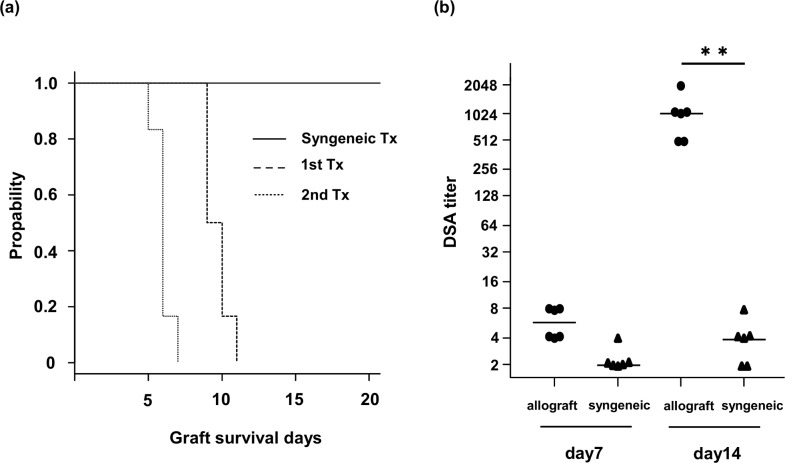
**Survival curve of transplanted skin grafts (a) and the kinetics of donor-specific antibody production in skin allografts and syngeneic graft recipients (b).** (a) Kaplan-Meier curve of skin graft survival for the first transplantation (1st Tx, dashed line), second transplantation (2nd Tx, dotted line) and syngenenic transplantation (syngeneic Tx, black line). Skin graft rejection was determined macroscopically when the graft reached a necrosis level of 90%. The median graft survival times of six mice per group were 9.5 (range 9–11) days for 1st Tx and 6 (range 5–7) days for 2nd Tx. Syngeneic grafts were not rejected for more than 21 days. (b) Production of donor-specific antibodies (DSA) in 1st Tx and syngeneic Tx recipients. On days 7 and 14 post-transplant, sera from each group (n = 6) were collected, and each dilution was assayed by flow cytometry for activity against donor thymocytes to determine the titer of each serum. Individual titers are shown with the median represented by the bar. **p < 0.01.

**Fig 7:**
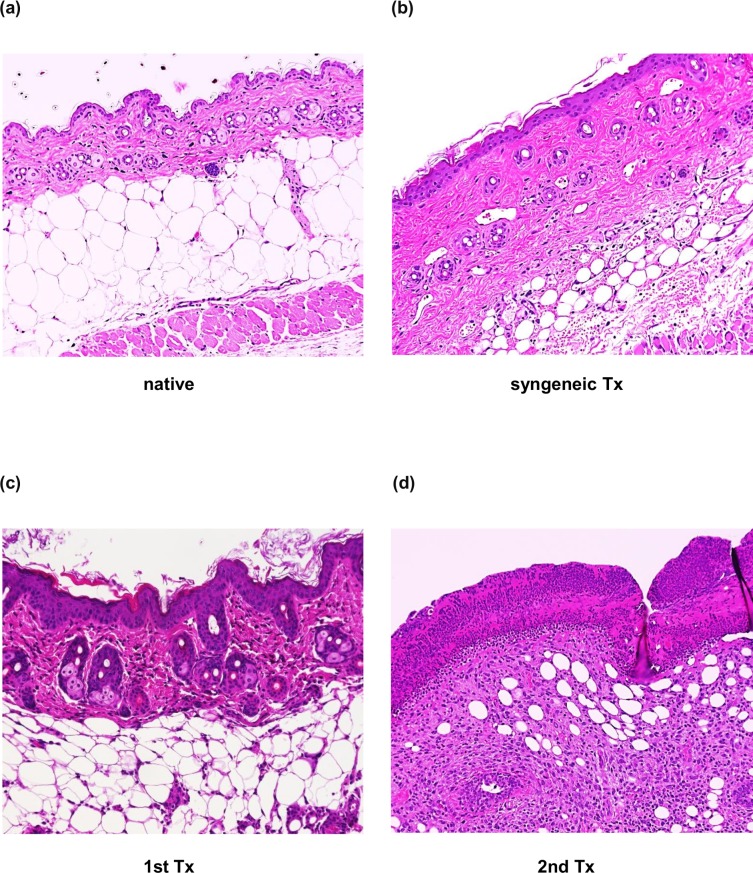
Histological examination of skin grafts at day 6 after transplantation. Skin graft specimens were obtained from a native donor graft (a), a syngeneic transplant recipient mouse (Syngeneic Tx, b), and allotransplanted mice with the first transplantation (1st Tx, c) and second transplantation (2nd Tx, d) on day 6. Paraffin sections were prepared and stained with hematoxylin and eosin. The syngeneic skin graft shows essentially normal histology without intra-epithelial infiltrates or apoptotic epithelial cells. The allograft of the 1st Tx shows epidermal thickening and mononuclear cell infiltration slightly into the graft dermis and hair follicles. Degeneration and necrosis are observed in the epidermis and skin appendages are almost lost in the allograft of the 2nd Tx. Magnification, ×200.

Based on the above clinical and in vitro data, we expected that the serum HSP90α level would increase in allotransplant recipients with AR and would be associated with the severity of rejection. Therefore, we analyzed the serum levels of HSP90α after skin transplantation in mice. The median survival of the skin allografts was 9.5 days after the first transplantation and it was 6.0 days after the second transplantation. In contrast, all syngenic skin grafts survived for > 21 days (Fig 6A). The titer of DSA in skin allograft recipients was significantly higher on day 14 after the first transplantation (p < 0.01, Fig 6B). Histological studies revealed mild infiltration of inflammatory cells in the dermal interstitium and epidermal hyperplasia in the first allograft on postoperative day 6 (Fig 7C). In the second allograft, most of the epidermal appendages became necrotic with massive inflammatory infiltrates in the dermal layer (Fig 7D). These histological findings and the high titer of DSA at the time of second transplantation are compatible with AABMR in the second allograft.

**Fig 8:**
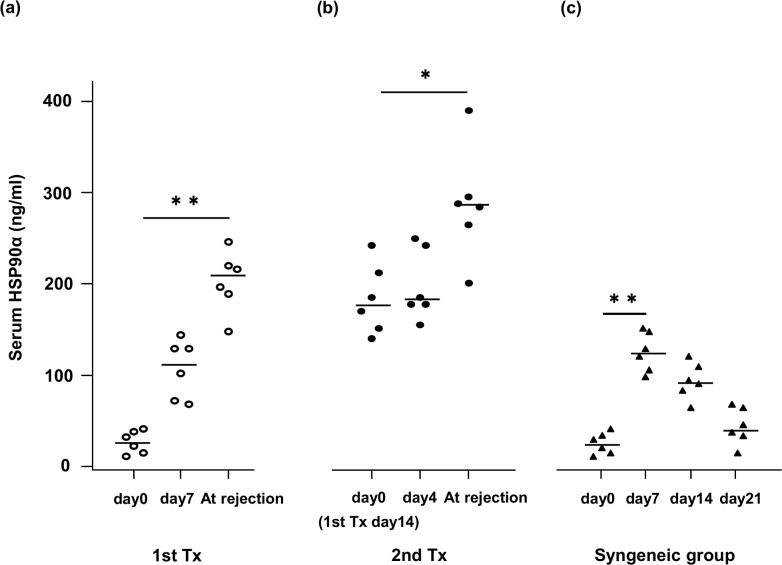
Scatter plots of serum HSP90α in skin transplantation of mice. Surviving mice were sacrificed and their sera were tested by ELISA. Each dot shows an individual result; bars represent the median. (a) The serum HSP90α level increased after fully mismatched skin transplantation. (b) In the second graft recipients, serum HSP90α was elevated at the time of rejection as compared to the time of rejection of the first graft. (c) In the syngeneic group, the serum HSP90α showed temporary elevation on day 7, and then gradually decreased to the baseline level. *p < 0.05, **p < 0.01.

The median serum HSP90α level was 24.33 ng/ml at baseline. It was elevated 4-fold at day 7 (median 114.6 ng/mL) and 7-fold at the time of rejection of the first graft (206.86 ng/mL, p < 0.01, Fig 8A). Moreover, serum HSP90α showed a further increase at the time of rejection of the second graft (178.22 ng/mL vs. 286.03 ng/mL, p < 0.05, Fig 8B). Even in the syngeneic skin transplants, the serum HSP90α level increased 4-fold from baseline at day 7 (24.33 ng/mL vs. 124.47 ng/mL, p < 0.01, Fig 8C). However, the level then decreased and was close to the baseline level at day 21 (43.05 ng/mL).

## Discussion

HSP90 is ubiquitously expressed in normal cells and contains two major cytosolic isoforms, HSP90α and HSP90β [11]. HSP90α is inducible under stress [11] and its expression level can be increased up to 10-fold [12]. In addition to heat shock, several physiological agents and conditions induce the expression of HSP90 in various cells [11]. Saito et al. [13] found that HSP90α was released into the serum and the levels correlated with the severity of the disease in SLE patients. In such patients, the HSP90 expression level is elevated in peripheral blood monocytes [3], and can be induced by IL-6 [14]. However, the mechanism of the elevation of serum HSP90α in SLE patients is still unknown. Patients with autoimmune vasculitis and mixed connective tissue disease also have higher serum HSP90α levels than healthy controls, whereas those with Sjögren's syndrome and Micklickz disease do not [13]. An active immune response may not always be associated with elevation of serum HSP90α. Cellular damage in immune cells, including lymphocytes and cells in other tissues, may also cause the release of HSP90α into the serum [13]. In our study, high serum HSP90α levels were obtained from patients whose graft vascular tissues were damaged by AR, such as those with AABMR and type II ATMR. We thus speculated that vascular cells damaged by allorejection could be a source of increased serum free HSP90α in kidney recipients. The results of our in vitro and in vivo studies supported this speculation. Severe damage to cultured endothelial cells induced by ligation with the anti-HLA antibody with complement attack resulted in abundant extracellular release of HSP90α. Furthermore, in the murine skin transplant model, the serum HSP90α level increased further at the time of second graft rejection that was compatible with AABMR, suggesting more severe vascular cell damage. In this study, the serum HSP90α level was high in two cases of Type IB ATMR, which must have had only interstitial infiltration of lymphocytes and tubulitis. It remains to be investigated what cells contribute to the serum level of HSP90α in kidney allograft rejection. Other cells in the kidney, including renal tubular cells and infiltrating immune cells, can also be a source of free HSP90α, although there is no report directly indicating that extracellular HSP90α is released from these cells. Through in vitro studies using mixed cultures of renal tubular cells and primed recipient's lymphocytes, we may be able to verify whether HSP90α is released by activated leukocytes or renal tubular cells with T cell infiltration. However, since it was impossible to collect the patients’ lymphocytes in the present study, we were not able to study this issue.

To date, several potential biomarkers of renal allograft rejection have been studied. The candidates include soluble adhesion molecules, cytokines, and the urokinase plasminogen activator receptor in the serum, expression of perforin, granzyme B, and FAS ligand in peripheral lymphocytes, adhesion molecules and complement C4d in urine, and the mRNA expression of granzyme B, perforin, and granulysin in urinary cells [15, 16]. Recently, some investigators reported specific microRNA in peripheral blood mononuclear cells or urinary cells as a potential biomarker of acute kidney rejection [17, 18]. However, no noninvasive marker other than serum creatinine is routinely used in the current clinical setting. The results of our study suggest that the serum HSP90α level can predict AR distinct from CIN before it is proven by biopsy. However, the HSP90α level in BKVN cases tended to be high compared to CABMR and CIN cases and those with no rejection. Since the sample size was small, further investigation with a larger sample size is needed. It might be useful for early detection of steroid-resistant rejection like AABMR, which can help us to make a quick decision to start anti-rejection treatment modalities besides steroids such as plasma exchange, intravenous immunoglobulin, and anti-CD20 antibody or lymphocyte-depleting antibody treatment. Moreover, our study showed that the serum HSP90α level decreased in kidney recipients after anti-rejection therapy, suggesting that a change in the value can be helpful for assessment of the effect of anti-rejection therapy, although the number of patients was small. On the other hand, extracellular HSP90α can be released during postoperative wound healing [19]. That may lead to an increase in the serum level of HSP90α as shown in our study of syngeneic skin transplantation in mice, although the level was not as high as that at the time of rejection. The serum HSP90α level is probably false positive early after surgery, so the kinetics of serum HSP90α in this period needs to be investigated.

This study has some limitations. It included only a small number of patients. Also, in addition to surgery, the primary disease causing chronic kidney disease and other concomitant disease may affect the serum HSP90α level. To validate the utility of serum HSP90α level as a marker of AR in kidney transplantation, further larger studies including patients with various backgrounds are needed. In addition, HSP90α secretion may potentially also be reduced by some agents. However, we could not examine the influence of agents on secretion of HSP90α in healthy individuals. Further research is needed to investigate the relationship between agents and the secretion of HSP90α in non-transplanted individuals.

In conclusion, this study has provided the first insights into the serum HSP90α level in kidney transplant patients with AR. The results of this study suggest that it is potentially a new biomarker of AR in kidney recipients.
